# Cerebral Oxygenation in Preterm Infants Developing Cerebral Lesions

**DOI:** 10.3389/fped.2022.809248

**Published:** 2022-04-12

**Authors:** Angelika L. Schwab, Benjamin Mayer, Dirk Bassler, Helmut D. Hummler, Hans W. Fuchs, Manuel B. Bryant

**Affiliations:** ^1^Division of Neonatology and Pediatric Intensive Care, Department of Pediatrics and Adolescent Medicine, University Hospital Ulm, Ulm, Germany; ^2^Institute of Epidemiology and Medical Biometry, Ulm University, Ulm, Germany; ^3^Neonatal Department, University Hospital Zurich, University of Zurich, Zurich, Switzerland

**Keywords:** preterm infant, intraventricular hemorrhage (IVH), periventricular leukomalacia (PVL), near-infrared spectroscopy (NIRS), cStO_2_, cerebral oxygenation, cerebral ischemia

## Abstract

**Background:**

We investigated the association between cerebral tissue oxygen saturation (cStO_2_) measured by near-infrared spectroscopy (NIRS) and cerebral lesions including intraventricular hemorrhage (IVH) and periventricular leukomalacia (PVL).

**Methods:**

Preterm infants <1,500 g received continuous cStO_2_ monitoring, initiated at the earliest time possible and recorded until 72 h of life. Mean cStO_2_ over periods of 5, 15, 30 min and 1 h were calculated. To calculate the burden of cerebral hypoxia, we defined a moving threshold based on the 10th percentile of cStO_2_ of healthy study participants and calculated the area under the threshold (AUT). cStO_2_ <60% for >5 min was regarded a critical event. The study was registered on clinicaltrials.gov (ID NCT01430728, URL: https://clinicaltrials.gov/ct2/show/NCT01430728?id=NCT01430728&draw=2&rank=1).

**Results:**

Of 162 infants (gestational age: mean 27.2 weeks, standard deviation 20 days; birth weight: mean 852 g, standard deviation 312 g) recorded, 24/12 (14.8%/7.4) developed any/severe IVH/PVL. Mean cStO_2_ was significantly lower in infants with IVH/PVL as well as severe IVH/PVL. In addition, we observed critical events defined by mean cStO_2_ over 5 min <60% in four infants with severe IVH/PVL during NIRS monitoring. AUT showed no statistically significant difference between outcome groups.

**Conclusion:**

These findings suggest that cStO_2_ is lower in infants developing IVH/PVL. This may be related to lower oxygenation and/or perfusion and implies that cStO_2_ could potentially serve as an indicator of imminent cerebral lesions.

## Introduction

Intraventricular hemorrhage (IVH) and periventricular leukomalacia (PVL) are among the most common cerebral lesions in preterm infants and represent important risk factors for adverse neurodevelopmental outcomes (1–3). According to Germany's quality report in 2015, the incidence of IVH and PVL in very low birth weight preterm infants <1,500 g are 4.0 and 1.5%, respectively (4).

Over 90% of IVHs develop within the first 3 days of life, and 50% within the first 24 h after birth (5). The exact time of occurrence of PVL is variable. PVL resulting from prenatal injury is typically observed early in life and often in combination with IVH (5, 6). The injury itself is thought to occur long before the cysts become detectable on ultrasound, but until now there is no reliable method to detect the exact timing of injury.

Hypoperfusion is considered a major risk factor in developing IVH or PVL, but the exact pathophysiological mechanisms still remain unknown (5, 7). In preterm infants, cerebral autoregulation is not fully matured leading to disturbances in cerebral blood flow as well as cerebral oxygenation. In order to monitor cerebral tissue oxygenation as a surrogate for cerebral perfusion, near-infrared spectroscopy (NIRS) as a non-invasive and easily applicable tool is increasingly used in the clinical setting. When peripheral oxygen saturation (SpO_2_) is available, cerebral fractional tissue oxygen extraction [CFTOE: (SpO_2_-cStO_2_)/cStO_2_] can show the relation between oxygen transport and consumption as an additional parameter.

NIRS was first described by Jöbsis and applied to neonates 7 years later (8, 9). It is used to monitor tissue oxygenation in various organs in a non-invasive manner. There are several devices on the market to monitor cStO_2_ in preterm infants. At the neonatal intensive care unit at Ulm University Children's Hospital, the ForeSight oximeter (Casmed, Branford, CT) is used.

Some but not all previous studies have already shown that preterm infants who developed IVH initially displayed lower cStO_2_ than unaffected preterm infants (10, 11). It has also been reported that a cycle of hypoperfusion and subsequent reperfusion could be detected in preterm infants who were diagnosed with IVH later in life (12, 13).

To our knowledge, no previous study has explored the potential relationship between adverse outcomes in preterm neonates and differences in cStO_2_ as well as the area under the threshold (AUT) established in healthy preterm infants using the ForeSight oximeter, but only using other oximeters (12, 14).

We aimed to assess the relationship between any IVH/PVL and cStO_2_ in the first 72 h of life. We investigated the correlation between IVH/PVL and mean cStO_2_ values as well as the AUT using a threshold developed in at risk infants that were considered healthy.

## Materials and Methods

We conducted a prospective observational study at the neonatal intensive care unit at Ulm University Children's Hospital between October 2010 and May 2014. The study was approved by the ethics committee (ethic vote 31/15) and was registered on clinicaltrials.gov (ID NCT01430728). Informed consent from the parents was waived as NIRS was part of standard monitoring at that time in infants considered at highest risk to develop intraventricular hemorrhage. Selection for NIRS monitoring was dependent on device availability, gestational age, birth mode, and risk of early onset bacterial infection.

### Subjects

Preterm infants with a birth weight below 1,500 g and who were subjected to NIRS-monitoring within 72 h of birth were enrolled. During the study period, 386 preterm infants with very low birth weight (<1,500 g) were born and received prospective treatment. Diagnostic or therapeutic clinical measures on the same or other preterm infants took precedence over observational NIRS monitoring.

### Data Acquisition and Diagnosis of IVH/PVL

Clinical data were collected from documented patient charts. IVH was defined according to the diagnostic criteria described by Papile et al. and the highest grade of hemorrhage observed during admission was used for analysis (15). As part of our minimal-handling protocol, aiming to reduce all procedures without immediate consequence, cranial ultrasound scans were performed in a restrained fashion, i.e., only on the fourth day of life, after 1 week and repeatedly before discharge from hospital.

### NIRS Measurement and Analysis

We obtained cStO_2_ data using a first-generation ForeSight cerebral oximeter during the first 72 h of life. The ForeSight cerebral oximeter measures absolute values of cerebral oxygen saturation using four LASER light sources with wavelengths of 690, 779, 808, and 850 nm and two detecting photodiodes. The depth of penetration is 2 cm (16). The ForeSight was chosen because it was the only device with FDA approval for absolute measurements at that time and because it was available in our unit from the utilization in previous research projects. We utilized non-adhesive neonatal sensors which was attached on both temporal sides of the infant's head with a cap. The position was regularly altered slightly during care to avoid skin damage.

NIRS monitoring initiated at the earliest time possible and recorded until 72 h of life. Yet for simplification of our analysis, the starting point was defined as the time of birth. CStO_2_ values were recorded every 2 s and were analyzed retrospectively using IBM software SPSS Statistics 21.0 following manual removal of artifacts. Artifacts were defined as values below 0% or above 92%. In addition, sequences of ≥20 consecutive equal values were deleted. Artifacts accounted for 8.4, 5.6, and 19.8% of cStO_2_, ECG heart rate, and peripheral oxygen saturation data, respectively.

We calculated mean averages over periods of 5, 10, 30 min and 1 h when there were values over a period of 30 s, 1, 2.5, and 5 min, respectively. We used repeated measures ANOVA to assess differences in cStO_2_ between outcome groups. Furthermore, an alternative threshold was defined as cStO_2_ mean average over 5 min <60%.

For the area under the threshold (AUT), we defined a baseline for every 2 s interval consisting of the 10th percentile of “healthy” preterm infants of our study population. The threshold was arbitrarily determined. The “healthy” control group contained cStO_2_ values of 105 preterm infants who didn't develop IVH/PVL, did not need resuscitation or catecholamines during the study period and who survived until discharge. AUT was calculated by considering each 2 s interval and if cStO_2_ measurement (%) was below the threshold, the difference between the cStO_2_ measurement and the threshold was calculated. AUT was then calculated using the difference multiplied by the duration of cStO_2_ measurements below the threshold in hours.


$$\begin{array}{l} AUT=\frac{1}{2}\sum_{i=1,\;\ldots ,n-1}^{\infty }\left(t_{i+1}-t_{i})\;x\;(C_{i+1}-C_{i}\right) \end{array}$$

i: Numbers of measurements t_i_: Time 1 t_i+1_: Time 2 C_i_: Variable value at time 1 C_i+1_: Variable value at time 2

## Results

### Study Population

**Figure 1:**
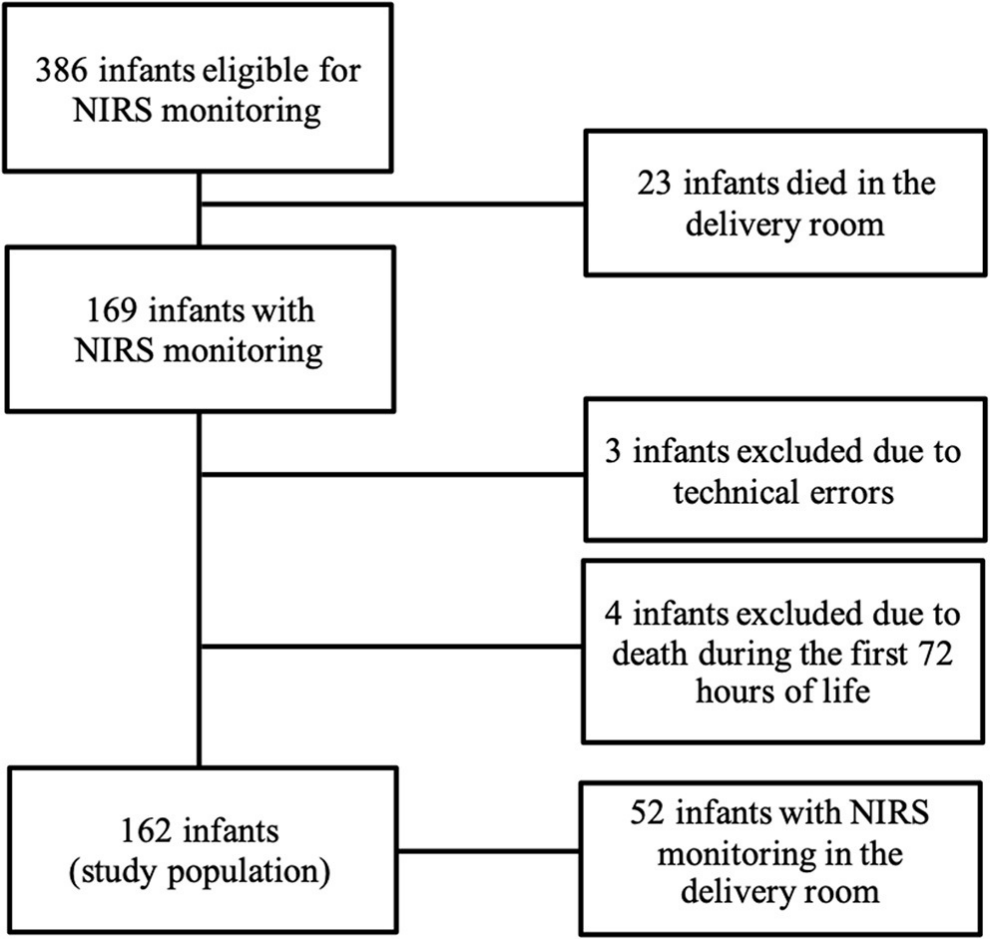
Patient flow diagram of preterm infants recorded study at the neonatal intensive care unit at Ulm University Children's Hospital during the study period between October 2010 and May 2014.

During the study period 386 preterm infants (gestational age ≥21 weeks, birth weight ≥200 g) were eligible for NIRS monitoring (gestational age: mean 27.2 weeks, standard deviation 20 days; birth weight: mean 852 g, standard deviation 312 g). Twenty-three infants >22 weeks died after a decision for comfort care in the delivery room. We obtained cStO_2_ measurements in 169 preterm infants. Due to technical errors, three infants were excluded. Four additional infants were excluded due to death during the first 72 h of life. Our study population thus consisted of 162 preterm infants. Out of the 162 infants enrolled, 24 (14.8%) developed cerebral lesions, among whom 2, 10, 4, and 6 developed grade 1, 2, 3, and 4 hemorrhage, respectively. In 3 infants cystic PVL was detected, 2 of these had no IVH. Severe IVH/PVL was defined as IVH grade III/IV or PVL. On average, cerebral lesions were detected after 6 and 7 days after birth, about 62.5% of cerebral lesions were diagnosed at the first ultrasound in the first 4 days after birth. A patient flow diagram is shown in Figure 1. Demographic characteristics and clinical data are shown in Table 1.

**Table 1:** Demographic characteristics and clinical data in outcome groups.

**Variable**	**No IVH/PVL (*n* = 138)**	**IVH/PVL (*n* = 24)**	***P*** **-value**
Gestational age in weeks^b^	27.3 ± 20 days	25.9 ± 15 days	0.010^+^
Birth weight in g^b^	872 ± 308	721 ± 319	0.029^+^
Male gender^a^	61 (44.2%)	16 (66.7%)	0.042*
C-section^a^	128 (92.8%)	17 (70.8%)	0.005*
Antenatal steroids (completed)^a^	86 (62.8%)	9 (37.5%)	0.020*
Intubation during first 72 h^a^	78 (56.5%)	20 (83.3%)	0.013*
Catecholamines during first 72 h^a^	37 (26.8%)	14 (58.3%)	0.002*
Starting time of catecholamines in hours after birth^b^	31.2 ± 14.8	29.0 ± 13.7	0.008^+^
Red blood cell transfusion^a^	39 (28.2%)	13 (54.2%)	0.012*
Mortality until discharge^a^	7 (5.1%)	5 (20.8%)	0.018*

a *Count (percentage of total)*.

b *Mean value ± standard deviation*.

+ *Independent samples t-test*.

* *Chi^2^-test. IVH, intraventricular hemorrhage; PVL, periventricular leukomalacia*.

### NIRS Measurements

**Figure 2:**
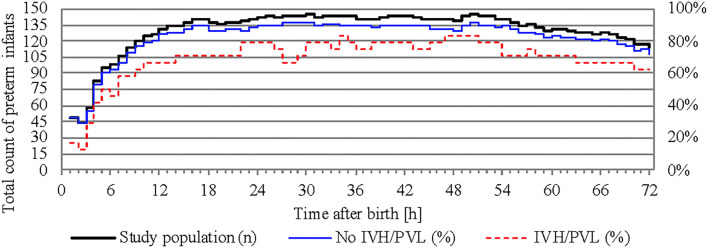
Total count of preterm infants recorded over time and percentage of calculated mean cStO_2_. The study population is represented on the left y-axis, outcome groups are represented on the right y-axis.

**Figure 3:**
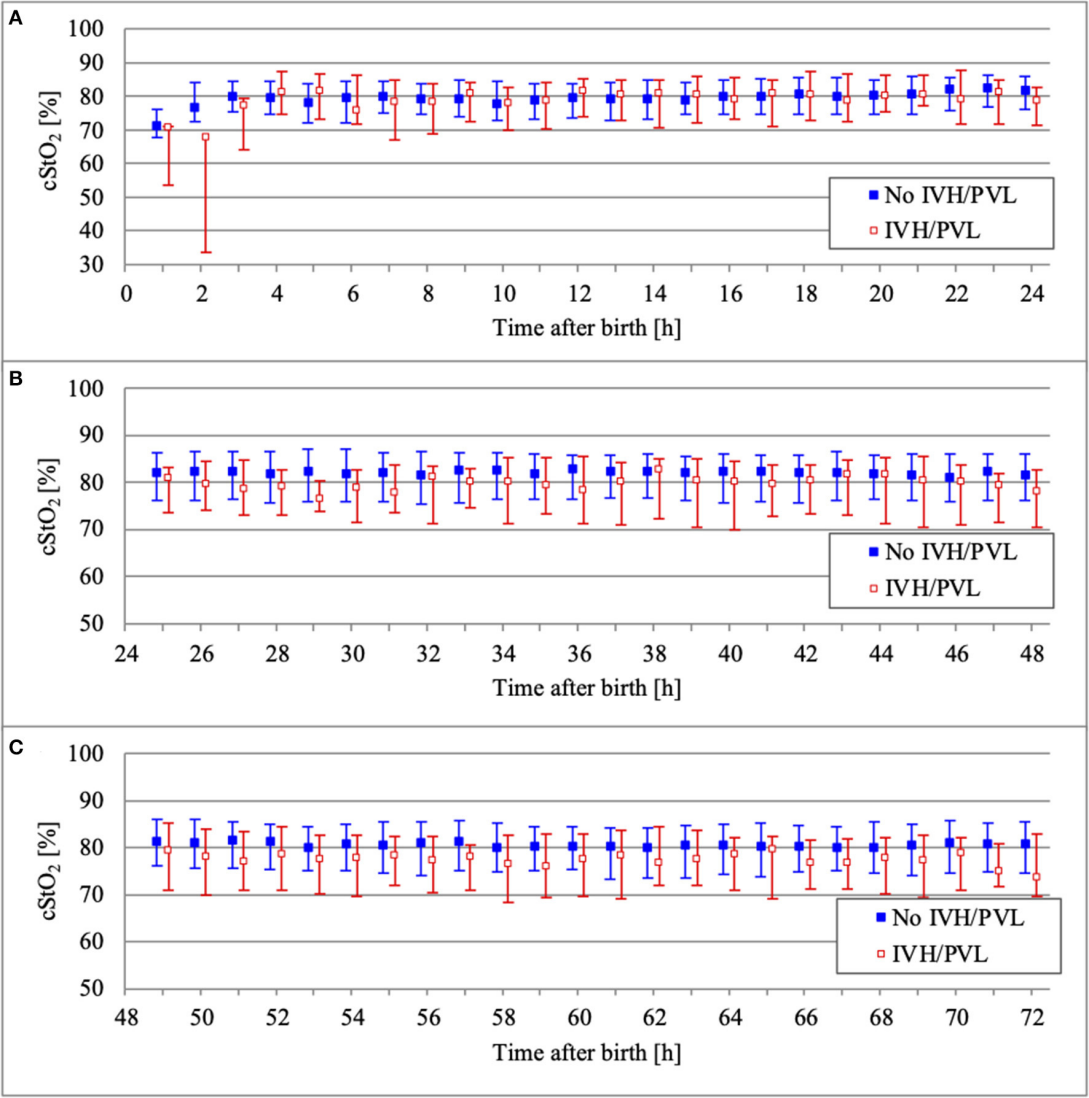
Comparison of cStO_2_ (median and interquartile range over 1 h) between outcome groups on the first day of life **(A)**, on the second day of life **(B)**, and on the third day of life **(C)**.

NIRS monitoring was started in the delivery room in 52 infants. The median age of our study population at the start of NIRS measurement was 3.2 h (interquartile range 0.2–8.0 h). Overall, the missing values for cStO_2_ amounted to 29.5% (36.4% for infants with IVH/PVL and 28.4% for infants without IVH/PVL). Hourly mean values of cStO_2_ could be obtained for 80.3% of the total 72 h in infants without IVH/PVL and for 70.8% in infants with IVH/PVL (Figure 2). Mean average of cStO_2_ over 1 h in both outcome groups is presented in Figure 3. Preterm infants with IVH/PVL had lower mean cStO_2_ than preterm infants without IVH/PVL, with the largest difference in cStO_2_ observed in the first 3 h of life. Throughout the remainder of the first day of life, mean cStO_2_ of both outcome groups converged. After 22 h, preterm infants with IVH/PVL once again had lower mean cStO_2_. Overall, median cStO_2_ over 72 h in preterm infants with IVH/PVL was 79.2% (interquartile range 71.1–83.6%) and lower than in preterm infants without IVH/PVL (median cStO_2_ 81.0%, interquartile range 75.2–85.4%).

**Figure 4:**
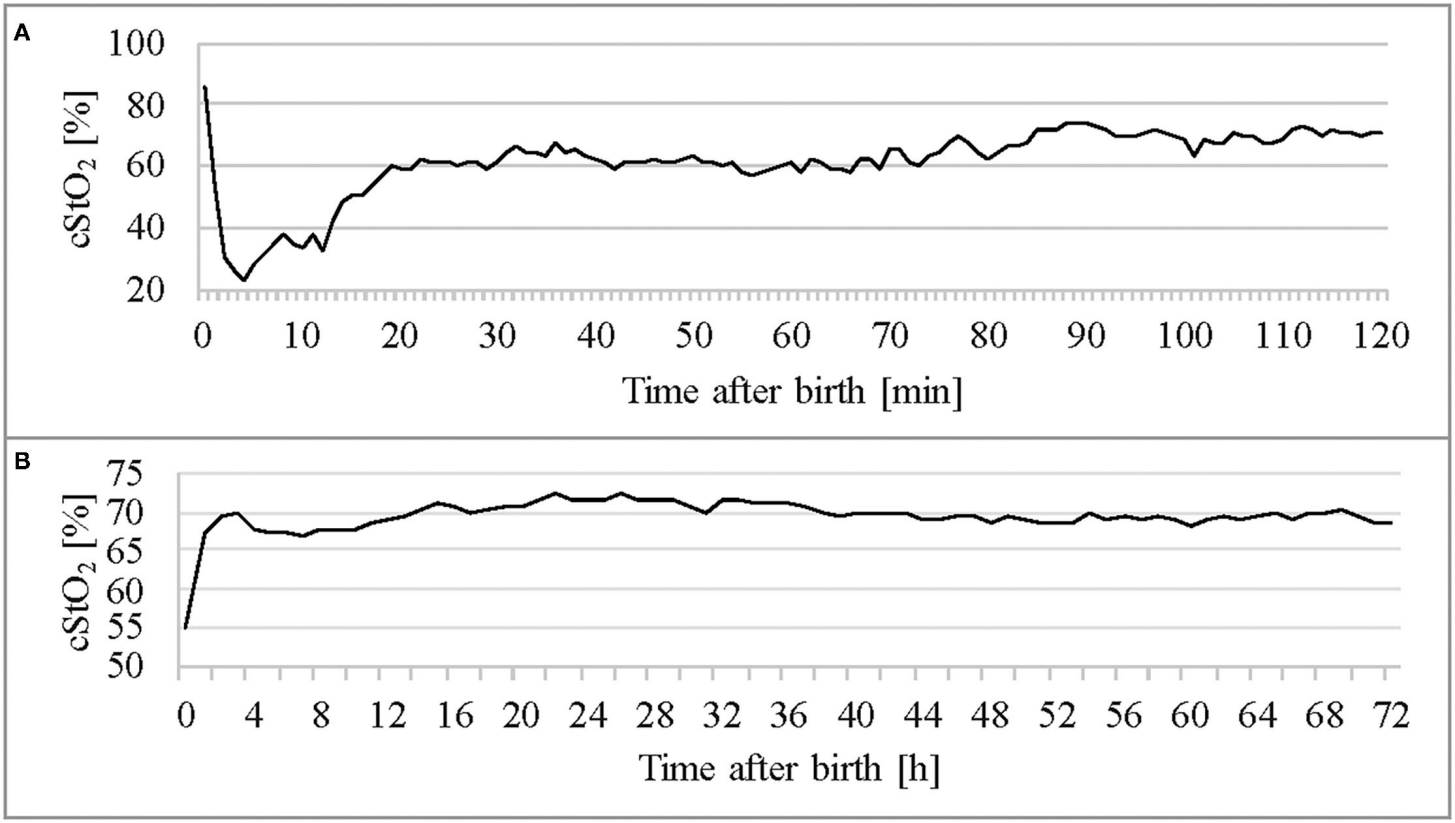
Thresholds of the healthy control group (*n* = 105) throughout the first 2 h **(A)** and in the first 3 days of life **(B)**.

Figure 4 shows per-minute mean thresholds for healthy preterm infants in the first 2 h of life (Figure 4A) and the hourly thresholds which were obtained by calculating the mean cStO_2_ for each 1-h period (Figure 4B). In the first 3 min cStO_2_ values were obtained of <10 infants, in the first minute cStO_2_ values were obtained of one infant with a mean cStO_2_ of 85%. After the first 19 min after birth the curve reaches a plateau. Tables 2, 3 show differences in mean cStO_2_ between IVH groups. Infants who developed IVH/PVL had a lower cStO_2_ in all computed mean averages. In patients with IVH/PVL, cStO_2_ was 2.4 percentage points lower than in those who did not develop cerebral lesions. Infants who developed severe IVH/PVL had a lower cStO_2_ in mean averages over 5 and 15 min. The difference in cStO_2_ amounted to 0.5 percentage points. The aforementioned discrepancies of total cStO_2_ were shown to be statistically significant. Regarding AUT we did not find a statistical relevant difference between outcome groups (Table 4).

**Table 2:** ANOVA with a mixed linear model.

**cStO_**2**_: mean average over**	**Estimates [%] for mean cStO_**2**_ (positive values indicate higher values in subjects without IVH/PVL)**	**95% confidence interval**	***P*** **-value**
1 h	2.41	2.00–2.86	<0.001
30 min	2.38	2.06–2.70	<0.001
15 min	2.37	2.14–2.61	<0.001
5 min	2.40	2.25–2.54	<0.001

*Outcome variable: mean averages of cStO_2_. Independent variable: IVH/PVL. cStO_2_, cerebral tissue oxygen saturation; IVH, intraventricular hemorrhage; PVL, periventricular leukomalacia*.

**Table 3:** ANOVA with a mixed linear model.

**cStO_**2**_: mean average over**	**Estimates [%] for mean cStO_**2**_ (positive values indicate higher values in subjects without severe IVH/PVL)**	**95% confidence interval**	***P*** **-value**
1 h	0.38	0.25–1.02	0.238
30 min	0.40	0.06–0.86	0.090
15 min	0.51	0.17–0.85	0.004
5 min	0.50	0.29–0.70	<0.001

*Outcome variable: mean averages of cStO_2_. Independent variable: severe IVH/PVL. cStO_2_, cerebral tissue oxygen saturation; IVH, intraventricular hemorrhage; PVL, periventricular leukomalacia*.

**Table 4:** Area under the threshold for cStO_2_ in the first 12 and 72 h of life.

**cStO_2_**	**No IVH/PVL (*n* = 138)**	**IVH/PVL (*n* = 24)**	***P*** **-value**
AUT 72 h	4.4 (0.7–20.0)	6.8 (0.7–80.1)	0.351
AUT 12 h	0 (0–1.1)	0.1 (0–1.8)	0.597

*AUT, area under the threshold; cStO_2_, cerebral tissue oxygen saturation; IVH, intraventricular hemorrhage; PVL, periventricular leukomalacia*.

**Figure 5:**
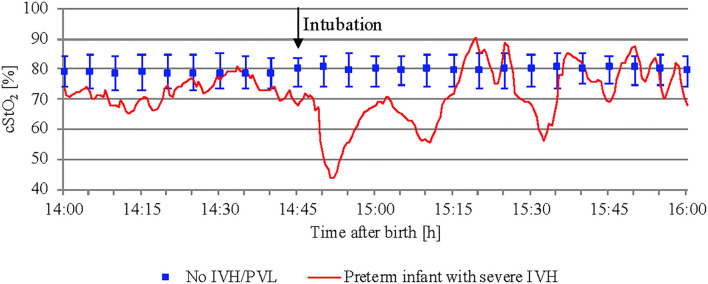
Critical event with cStO_2_ <60% in a preterm infant with severe IVH after intubation. In addition, preterm infants without IVH/PVL are presented (median and interquartile range).

While studying mean cStO_2_ over 5 min periods in preterm infants with severe IVH or PVL, critical events with a significant drop in cStO_2_ <60% were observed in four out of 12 infants. Two of them developed long episodes over 3–6 h with cStO_2_ <60%. Subsequently, both died within the first 72 h after birth. Figure 5 shows an example of how the cStO_2_ curve was affected during a critical event with cStO_2_ <60%. The other two preterm infants showed four short events with cStO_2_ <60% each.

**Figure 6:**
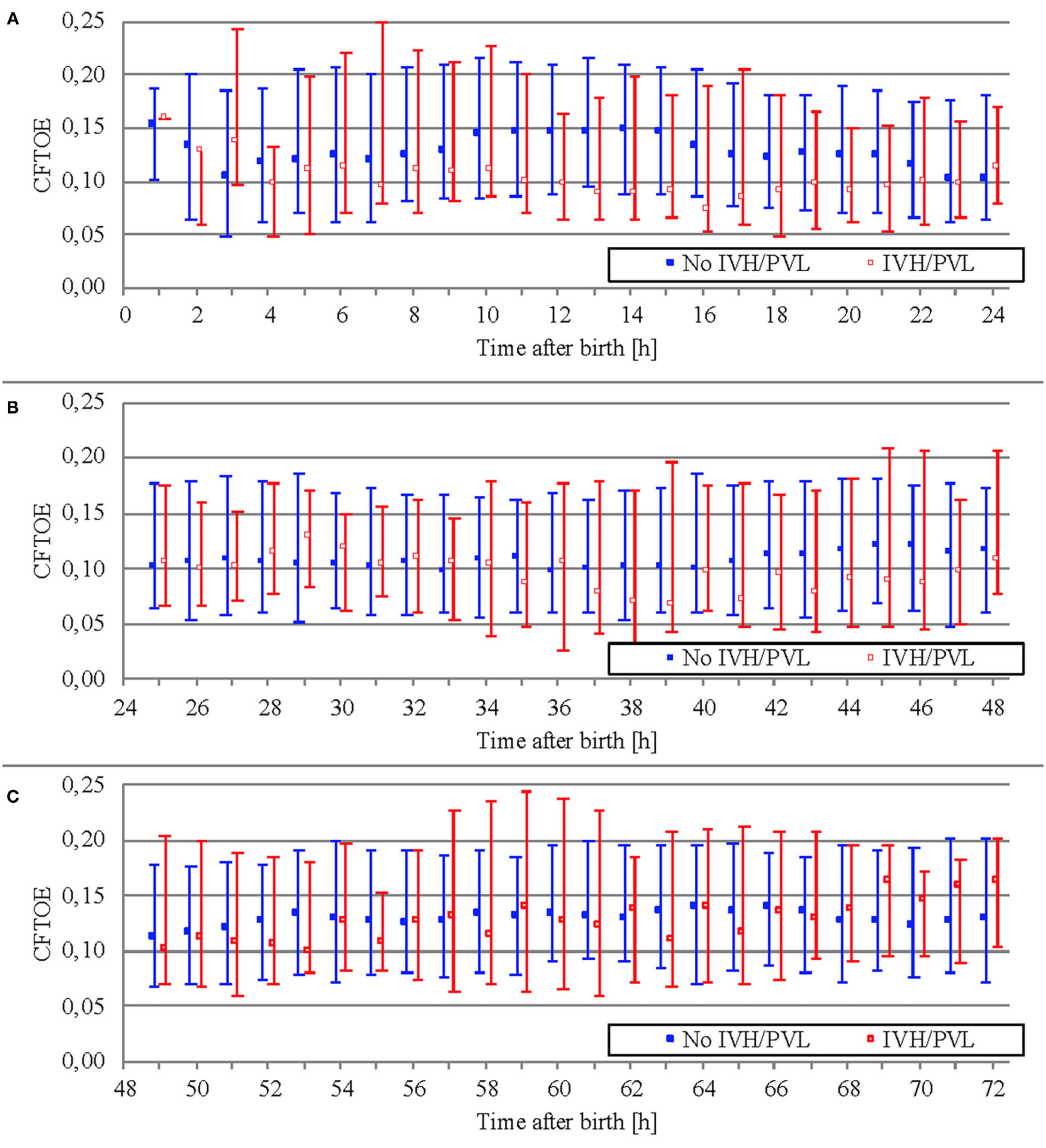
Comparison of CFTOE (median and interquartile range over 1 h) between outcome groups on the first day of life **(A)**, on the second day of life **(B)**, and on the third day of life **(C)**.

Data on CFTOE is shown in Figure 6. We found a minimal significant difference of 0.005 between both groups regarding mean averages (Table 5), but no significant difference regarding AUT (Table 6).

**Table 5:** ANOVA with a mixed linear model.

**CFTOE: mean average over**	**Estimates [%] for mean CFTOE (positive values indicate higher values in subjects without IVH/PVL)**	**95% confidence interval**	***P*** **-value**
1 h	0.004	−0.001 to 0.009	0.086
30 min	0.005	0.001–0.008	0.012
15 min	0.005	0.002–0.007	<0.001
5 min	0.004	0.002–0.005	<0.001

*Outcome variable: mean averages of CFTOE. Independent variable: IVH/PVL. CFTOE, cerebral fractional tissue oxygen extraction; IVH, intraventricular hemorrhage; PVL, periventricular leukomalacia*.

**Table 6:** Area under the threshold for CFTOE in the first 12 and 72 h of life.

**CFTOE**	**No IVH/PVL (*n* = 138)**	**IVH/PVL (*n* = 24)**	***P*** **-value**
AUT 72 h	0 (0.03–0.13)	0.06 (0–0.26)	0.383
AUT 12 h	0 (0–0.01)	0 (0–0.01)	0.815

*CFTOE, cerebral fractional tissue oxygen extraction; IVH, intraventricular hemorrhage; PVL, periventricular leukomalacia*.

## Discussion

In this study we showed that preterm infants with very low birth weight who developed (severe) IVH/PVL had a lower mean cStO_2_ in the first 72 h of life. Our findings confirm the results of previous studies that demonstrated an association between hypoperfusion and IVH (10, 11, 17). In the present study, however, AUT did not differ between outcome groups. By contrast, a recent study of 44 infants <28 weeks using the NIRO-200NX NIRS monitor by Ng et al., testing different thresholds for cStO_2_ ranging from 62 to 80%, reported that infants who developed IVH had a higher AUT during the first 24 h of life (14). Moreover, the time spent under different thresholds (60–67%) was also higher for infants with IVH. These opposing results can possibly be explained by differences in study design. Ng et al. chose a static threshold definition, while we employed a dynamic approach and calculated a specific threshold for every 2 s interval. Furthermore, the study population of Ng et al. had a lower gestational age compared to our population (No IVH group: 25.7 vs. 27.3 weeks). The observation time was shorter (24 vs. 72 h) and NIRS measurement started later (median age 5.5 h, interquartile range 3.1–12.6 h vs. median age 3.2 h, interquartile range 0.2–8.0 h).

Impaired cerebral autoregulation is a risk factor for IVH/PVL (18, 19). While a number of characteristics such as low gestational age, cardiac dysfunction and asphyxia are associated both with poor autoregulation and with IVH/PVL (20, 21), there is no bedside tool available to reliably diagnose the margins and the quality of cerebral autoregulation in a particular infant in daily routine care (7). While some infants with few risk factors develop IVH/PVL, others with a substantial amount of such risk factors don't. Measurement of cStO_2_ is a potential way to detect episodes of impaired autoregulation (12, 22, 23). Although we were unable to define a strict threshold to predict or rule out IVH, we may have missed the potential to detect impaired autoregulation, which would require a more thorough analysis of cStO_2_, CFTOE, and arterial blood pressure.

Moreover, variations in cerebral oximeter readings among devices (NIRO vs. ForeSight) might account for the difference in outcomes. A study by Kleiser et al. comparing various NIRS devices on a liquid phantom provided a table allowing for conversion of cStO_2_ values (24). Using the hypoxic threshold of 71% used by Ng et al. and the table by Kleiser et al. we calculated an equivalent hypoxic threshold of 73.3% and computed AUT over the first 24 h of life. However, this approach revealed no statistically significant differences between groups (AUT of infants with IVH/PVL: median 3.2, interquartile range 0.2–44.4, AUT of infants without IVH/PVL: median 3.2, interquartile range 0.4–18.5, *p*-value 0.905).

Our results regarding critical events with a significant drop in cStO_2_ <60% in infants with severe IVH/PVL suggest that time spent under a specific baseline causing a hypoperfusion-reperfusion-cycle can indeed be an important factor in the pathogenesis of IVH.

Gaps in NIRS recording accumulated to 28.2% (interquartile range 8.0–71.0%) of the total 72 h recording time. Hence, we analyzed these patients' paper charts, to check whether there were critical incidents during recording gaps during which cStO_2_ might also have been critically low. We found critical events in 7 out of 8 infants, which may have led to a significant drop in cStO_2_. Seven patients were intubated in the delivery room without NIRS recording, one of them was also resuscitated using chest compressions. Six developed hypotensive episodes which required catecholamine during which NIRS-monitoring was paused. Only one infant without a significant cerebral desaturation did not present critical events of any kind in the patient chart during recording gaps. The patient was diagnosed with PVL but without IVH. This infant suffered from early onset sepsis after preterm rupture of membranes and amniotic infection syndrome, but it was circulatory stable at all times since birth.

### Limitations

This study has several limitations. Firstly, we selected preterm infants for NIRS-monitoring not population-based or randomly, but according to their perceived risk for developing IVH/PVL. As a result, our study population was more premature and had a lower birth weight than the total population of very-low-birthweight-infants from October 2010 to May 2014. Because of our biased sample the representativity is uncertain. The second limitation consists in the small number of cases. While it is strength of our study, that recordings were started in some infants as soon as in first minutes of life, we still have large recording gaps. For half of all infants, we missed the first 3 h of life. There are reasons to believe that these gaps are more likely to appear during critical conditions, when the main focus of the team in charge was to stabilize the infant and not to keep the NIRS recording working. As a consequence, we might have missed the most critical situations during our recordings. While it is possible that even short but profound episodes of cerebral ischemia may lead to IVH, the NIRS threshold below which the risk for such ischemia is increased is not known. Our approach to use a healthy population to define such a threshold will not necessarily lead to identification of an individual and situational threshold for each infant. Following the hypothesis of a sequence of hypoperfusion and hyperperfusion may lead to IVH, synchronization of all infants by time of birth for development of cStO_2_ percentiles is inferior as compared to an approach synchronizing time of occurrence of the hemorrhage, because at the very same postnatal age, one group of infants might be still in the phase of hypoperfusion while another group might already be in the phase of hyperperfusion with the consequence of averaged mean values. Another limitation is the isolated evaluation of cStO_2_ data. The simultaneous recording of cStO_2_, SpO_2_, transcutaneous pCO_2_, heart rate, and arterial blood pressure gives the opportunity to examine interdependencies between these parameters and to evaluate the quality and margins of cerebral autoregulation. Factors associated with impairment of cerebral autoregulation widely overlap with factors associated with IVH/PVL. Thus, it may be insightful to study our data with a focus on interdependence and autoregulation in order to detect patterns that are predictive of IVH/PVL. Finally, NIRS measurement was only recently implemented as part of our standard monitoring for preterm infants and is therefore prone to technical errors and handling issues by the staff leading to artifacts and recording gaps.

## Conclusion

Our study demonstrates that preterm infants <1,500 g with IVH or PVL had lower cStO_2_ during the first 72 h of life compared to healthy preterm infant controls. The largest difference in NIRS-monitored cerebral oxygen saturation was seen during the first 3 h of life. In addition, we observed critical events defined by mean cStO_2_ over 5 min <60% in four preterm infants with severe IVH/PVL (*n* = 12) during NIRS monitoring. For seven of the remaining infants, we could identify critical situations which may have led to a significant drop in cStO_2_ during recording gaps while analyzing patient charts. These findings suggest that cerebral perfusion may be decreased before development of IVH/PVL. However, further studies are required to define normal ranges of cStO_2_ and to assess specific interventions guided by NIRS-monitoring to reduce the risk for IVH/PVL in preterm infants.

## Data Availability Statement

The original contributions presented in the study are included in the article/supplementary materials, further inquiries can be directed to the corresponding author/s.

## Ethics Statement

The studies involving human participants were reviewed and approved by University of Ulm, Ethical Committee. Informed consent from the parents was waived as NIRS was part of standard monitoring at that time in infants considered at highest risk to develop intraventricular hemorrhage.

## Author Contributions

AS: data curation, investigation, formal analysis, and drafting the initial manuscript. BM: data curation and formal analysis. DB: formal analysis and supervision. HH: conception or design of the work, methodology, investigation, supervision, and resources. HF: conception or design of the work, methodology, and investigation. MB: conception or design of the work, methodology, data curation, investigation, formal analysis, and supervision. All authors: review and editing of the manuscript, final approval of the version to be published, and agreement to be accountable for all aspects of the work.

## Conflict of Interest

The authors declare that the research was conducted in the absence of any commercial or financial relationships that could be construed as a potential conflict of interest.

## Publisher's Note

All claims expressed in this article are solely those of the authors and do not necessarily represent those of their affiliated organizations, or those of the publisher, the editors and the reviewers. Any product that may be evaluated in this article, or claim that may be made by its manufacturer, is not guaranteed or endorsed by the publisher.
